# Cannabis Use from Early Adolescence to the Mid-Twenties in Children of Immigrant and Nonimmigrant Parents: Findings from a Prospective Longitudinal Cohort Study

**DOI:** 10.1007/s11469-024-01359-0

**Published:** 2024-07-03

**Authors:** Annekatrin Steinhoff, Laura Bechtiger, Kurt Birchler, Denis Ribeaud, Manuel Eisner, Boris B. Quednow, Lilly Shanahan

**Affiliations:** 1 University Hospital of Child and Adolescent Psychiatry and Psychotherapy, University of Bern, Bolligenstrasse 111, 3000 Bern, Switzerland; 2 Jacobs Center for Productive Youth Development, University of Zurich, Zurich, Switzerland; 3 Institute of Criminology, University of Cambridge, Cambridge, UK; 4 Psychiatric University Hospital Zurich, University of Zurich, Zurich, Switzerland; 5 Neuroscience Center Zurich, University of Zurich and Swiss Federal Institute of Technology Zurich, Zurich, Switzerland; 6 Department of Psychology, University of Zurich, Zurich, Switzerland

**Keywords:** Cannabis, Adolescence, Immigration, Longitudinal, Latent growth

## Abstract

This study compares the developmental course of cannabis use in adolescents with versus without an immigrant background. Data came from a Swiss prospective-longitudinal cohort study (*n* = 1445) with nine assessments between ages 7 and 24. Parents reported their immigration history; adolescents self-reported their past-year cannabis use five times between ages 13 (in 2011) and 24 years (in 2022). Latent growth curve models revealed a curvilinear increase in cannabis use, with a peak at age 20. Adolescents whose parents had immigrated showed a less steep increase in cannabis use during adolescence and a lower cumulative prevalence of cannabis use by age 24. Specific cultural and religious backgrounds were linked with lower odds of cannabis use. Interventions in early adolescence need to consider immigration, cultural, and religious backgrounds.

In Western countries, including the USA and European countries, immigrants are often in better health and live longer than nonimmigrants (Kaplan et al., 2004; Markides & Rote, 2015; Uretsky & Mathiesen, 2007). These findings are notable considering that immigration involves considerable stressors and adversities (Markides & Rote, 2015), which could induce poorer health. A similar phenomenon has emerged for adolescent substance use: In the USA and northern Europe, adolescent first- and second-generation immigrants (henceforth referred to as “immigrant youth”) tend to report lower rates of alcohol and illicit substance use than their nonimmigrant peers (Amundsen et al., 2005; Gfroerer & Tan, 2003). Considering that the prevalence of substance use typically increases rapidly during mid-adolescence until it peaks in early adulthood (Copeland et al., 2017; Shanahan et al., 2021; Steinhoff et al., 2022), insights into differences in the longitudinal course of substance use between groups with and without immigrant backgrounds could be important for informing preventions and interventions.

To date, the immigrant effect on adolescent substance use has not been studied from a developmental perspective, and the substantial heterogeneity within the immigrant population is rarely considered. When and why do differences in substance use between immigrant and nonimmigrant youth unfold across adolescence? What explains the differences between immigrant youth and their nonimmigrant peers, and within immigrant groups? Understanding these questions is especially important for cannabis use, for which legalization trials are underway in several Western countries.

## Cannabis Use in Switzerland and the Role of Immigration Backgrounds

Switzerland is a wealthy nation with high rates of immigration (Bundesamt fuer Statistik, 2019) and adolescent cannabis use (Quednow et al., 2022; Ribeaud, 2014; Shanahan et al., 2021; Ter Bogt et al., 2006). In our study’s sample — a largely representative community sample — more than one in two adolescents reported cannabis use by age 17; additional hair toxicology analyses indicated that the prevalence of *frequent* cannabis use was about 14% at age 20 (Steinhoff et al., 2023). Cannabis use is currently illegal in Switzerland, but de facto decriminalized, given that penalties for use and possession are mild, and open cannabis use is often tolerated by law enforcement (Hehli, 2017; Zobel & Marthaler, 2016). Legalization trials have been underway in several Swiss cities since 2023. Many Swiss youth perceive cannabis as a relatively safe drug, to an even greater extent than youth in other European countries (Andersson et al., 2009).

In several studies, immigrant youth in Switzerland had lower rates of alcohol use than their nonimmigrant peers (Campisi et al., 2017; Hüsler & Werlen, 2010). Lower cannabis use has been shown for immigrant adults (Gmel et al., 2018; Vogel et al., 2019), and for 11–20-year-olds in one cross-sectional study (Hüsler & Werlen, 2010). Yet, in another cross-sectional study of 15–24-year-olds, immigrant youth and their peers did not differ in their cannabis use (Campisi et al., 2017). Since these studies examined different age-groups, it is possible that differences in the findings arose because the immigrant effect changes (e.g., decreases) with age.

## Factors Associated with Lower Cannabis Use among Immigrants

There are several potential reasons why cannabis use may be lower among immigrant youth; when considering these reasons, the heterogeneity of the immigrant population must be considered. First, region or country of origin likely plays a role. Cannabis use is illegal in many countries, viewed as dangerous, and prosecuted with severe legal penalties (Andersson et al., 2009; Sznitman, 2007; Zobel, 2017; Zobel & Maier, 2018); immigrants may bring these views with them. Indeed, rates of cannabis use are lowest in low income countries with punitive laws for cannabis use/possession (e.g., some Asian and African countries) (Degenhardt et al., 2008; Shi et al., 2015). As they arrive in their new country, immigrants tend to view the local laws as legitimate and are also unlikely to want to break these laws for fear of deportation (Piquero et al., 2016). Thus, laws in the country of origin and respect for the laws in the new country may combine to result in lower rates of cannabis use among youth.

Religion is another potential reason. Religiosity is declining among youth in Switzerland, but many immigrants arrive with stronger affiliations to religions. For example, many families from the former Balkan states are Muslims, and Islam taboos intoxication and forbids alcohol. Indeed, Muslim youth tend to drink no or less alcohol than non-Muslim youth (Amundsen et al., 2005), and Muslim and Christian youth are less likely to use cannabis than youth with no or another religious affiliation (Abebe et al., 2015).

Another important source of heterogeneity within the immigrant population is the length of residency and acculturation. Research from the USA reports that longer residency (and, thus, perhaps, acculturation to US culture) is associated with poorer health among immigrants (Kaplan et al., 2004; Uretsky & Mathiesen, 2007). Length of residency may also influence attitudes toward the use of illegal drugs, including cannabis (Amundsen et al., 2005; Lukash & Killias, 2018). For example, more accultured families in Switzerland may view cannabis use as more normative, and give their adolescents more liberties, meaning that these adolescents are more fully integrated into the opportunity structures of the Swiss drug environment (e.g., easy access to cannabis and unsupervised time away from home at night).

Altogether, immigrant parents need to navigate between values and developmental goals for their children according to their cultures of origin and religious beliefs (i.e., their own previous socialization) and beliefs, values, and common practices (e.g., regarding values transmitted and liberties granted to their adolescent offspring) in the new country (Motti-Stefanidi, 2018). The tendency towards adherence to the new country’s common practices and norms may generally increase with longer residency. Although adolescents become increasingly independent from their families as they mature, family socialization in childhood and adolescence as shaped by parental cultural and religious backgrounds is likely to impact adolescent behavioral development, including substance use, until adulthood (Roche et al., 2008).

## The Current Study

Our aim was to contribute to a better understanding of how socio-demographic and cultural factors are related to adolescent cannabis use. Novel insights into factors explaining the heterogeneity of cannabis use among adolescents are needed to facilitate profound evaluations of the generalizability of previous knowledge and to identify targets of prevention mechanisms that are relevant in specific social and cultural contexts. We examined (1) whether parental immigration background is associated with differences in the longitudinal course of cannabis use from early adolescence to the early twenties, and (2) whether region of origin, religious background, and length of residency explain differences in cannabis use between immigrant and nonimmigrant youth.

Since some previous research, including based on our community sample, has shown a higher prevalence of substance use in male adolescents than female adolescents (Copeland et al., 2017; Quednow et al., 2022; Ribeaud, 2014; Shanahan et al., 2021) and gender roles may also differ in some immigrant families, we also tested immigrant differences by sex. Furthermore, while many adolescents try cannabis only once or a few times, others use cannabis more regularly and some become dependent. Research on alcohol use has reported that immigrant differences are larger for *any* use than for *frequent* use (Amundsen et al., 2005). These findings suggested that immigrant youth may have a higher threshold for initiating substance use but not necessarily for progressing toward more regular use. To our knowledge, comparisons between immigrant and nonimmigrant youth have not examined frequent cannabis use, and we aim to fill this gap in research by conducting our analyses for (a) any cannabis use and (b) frequent cannabis use.

We use data from the ongoing prospective-longitudinal *Zurich Project on Social Development from Childhood to Adulthood* (*z-proso*; (Ribeaud et al., 2022)). This is a community study of adolescents growing up in and around Zurich, Switzerland’s largest city. With its largely representative sample, of which more than two-thirds had an immigrant parent, and repeated assessments of cannabis use from early adolescence to young adulthood, the study offers unique opportunities to provide new insights into the links between migration backgrounds and cannabis use across adolescence.

## Methods

### Data

Participants in z-proso were selected through a cluster-stratified randomized sampling approach. In 2004, a sample of 1675 children from 56 primary schools was randomly selected from 90 public schools in the city of Zurich. Stratification was performed considering school sizes and socioeconomic background of school districts. The sample was largely representative of first-graders attending public school in the city of Zurich. After the first assessment, at age 7 years, the children were assessed again in two-to-three-year-intervals until they reached age 24 years, in 2022, when the most recent assessment was carried out. Overall, participants were assessed nine times (i.e., at ages 7, 8, 9, 11, 13, 15, 17, 20, and 24 years). Cannabis use and its frequency, which are at the core of the investigation presented here, were assessed five times between ages 13 and 24.

The current study uses data from *n* = 1445 youth, for whom data on both parents’ countries of origin was available. Consistent with Switzerland’s immigration policies and the city’s diverse population, parents of participants had been born in >80 different countries; 76% of adolescents grew up with at least one immigrant parent. Most adolescents were born in Switzerland (91%); the remainder had arrived in Switzerland by 1^st^ grade, when the sample was recruited.

Adolescents provided written consent for their study participation. Until age 15, parents could opt their child out of the study. Data were collected in groups of 5–25 participants in classroom-based settings with paper and pencil questionnaires up to age 17, and in a computer laboratory setting with computer-administered surveys at ages 20 and 24. Survey completion typically took approximately 60–90 min. Online completion of the survey was possible at ages 20 (3% of participants completed the survey online) and 24 (15% of participants). During the first four assessments (ages 7–11), the primary caregiver was interviewed (computer-assisted) at home. Participants received a cash incentive, which increased from $30 at age 13 to $75 at age 20 and $150 at age 24 for on-site participation ($100 for online participation). The study is consistent with national and international ethics standards and was approved by the Ethics Committee of the Faculty of Arts and Social Sciences of the University of Zurich.

### Variables

Past-year *cannabis use* was self-reported at the ages of 13, 15, 17, 20, and 24 years. The participants rated their frequency of cannabis use on a six-point scale from 1 = “never” to 2 = “once,” 3 = “2–5 times,” 4 = “6–12 times (monthly),” 5 = “13–52 times (weekly),” and 6 = “53–365 times (daily).” We created dummy variables indicating whether the adolescents reported *any* cannabis use in the previous year at a given assessment, and whether they ever reported any cannabis use between ages 13 and 24 (i.e., cumulative prevalence). Similarly, we created dummy variables indicating *frequent* (i.e., at least weekly to daily) cannabis use during the previous year and across the study period. This categorization was based on previous research showing that reporting at least weekly cannabis use during one’s teenage years is associated with an increased risk of a variety of subsequent social and psychological impairments and other substance use in adulthood (Patton et al., 2005; Shanahan et al., 2021; Silins et al., 2014).

*Migration background.* Most immigrant youth in our sample were 2^nd^ generation immigrants (i.e., born in Switzerland to immigrant parents). We distinguished three groups of adolescents: (1) both parents born in Switzerland (*n* = 341), (2) one immigrant parent (i.e., one parent born abroad, the other one born in Switzerland; *n* = 388), and (3) two immigrant parents (i.e., both parents born abroad; *n* = 716).

*Parental country of birth* was reported by the primary caregiver at the second assessment (age 8) and by adolescents at ages 13 and 15, referring to the biological parents. We used the adolescents’ reports because these variables had fewer missing data than the parental reports. When both adolescent and parent reports were available, agreement on the parental country of birth was high. In our analyses, Switzerland was the reference country of origin. Other countries were combined to represent five major regions of origin (i.e., former Yugoslavia, Africa, Asia, Europe [excl. former Yugoslavia], Latin America) and an “other” category, including, for example, the USA, Canada, and New Zealand. We created dummy variables indicating (a) if any parent was born in a specific region and (b) combinations of regions of origin among parent pairs. For version b, two “mixed” categories were created (i.e., “mixed including one nonimmigrant parent” and “mixed with two immigrant parents”). Version a (i.e., any parent) was used only in descriptive statistics for group characterizations; version b (i.e., parent pairs) was used in the analyses of group differences in cannabis use. This was done to allow for a maximally clear differentiation between regional backgrounds.

*Religious background.* Participants were asked to report their religious denomination irrespective of whether they were practicing at age 13 (or at age 15, for those who had not participated at age 13).

*The length of residency* in Switzerland was parent-reported at the first assessment. Specifically, the primary caregiver reported when they and their partner first came to live in Switzerland, if applicable (range: 1955–2005). From this information, we derived a variable that counted the years between entering Switzerland as an immigrant and the first interview, thus representing the years of residency in Switzerland before the child’s school entry. If data on both caregivers was available, we computed a mean score of their lengths of residency; if data on only one caregiver’s length of residency was available or only one caregiver had immigrated, this information was used (range: 0–49 years, M = 14.76, SD = 7.96). Consistent with prior research (Kaplan et al., 2004; Uretsky & Mathiesen, 2007) and to allow for a comparison between different lengths of residency versus having two nonimmigrant parents (i.e., categorical information), we created dummy variables. Specifically, different lengths of residency were grouped into (a) 0–5 years, (b) 6–10 years, (c) 11–15 years, (d) 16–20 years, and (e) 21 and more years.

We adjusted for the households’ socio-economic status as assessed using the parents’ *International Socio-Economic Index of Occupational Status* (ISEI (Ganzeboom et al., 1992)). This is an internationally comparable index of socio-economic status based on occupation-specific income and the required educational level, with scores ranging from 16 (e.g., unskilled worker) to 90 (e.g., judge). We also adjusted for the *primary caregiver’s age* at the first assessment (M = 37.02, SD = 5.37).

## Analytical Strategy and Missing Data

**Fig. 1:**
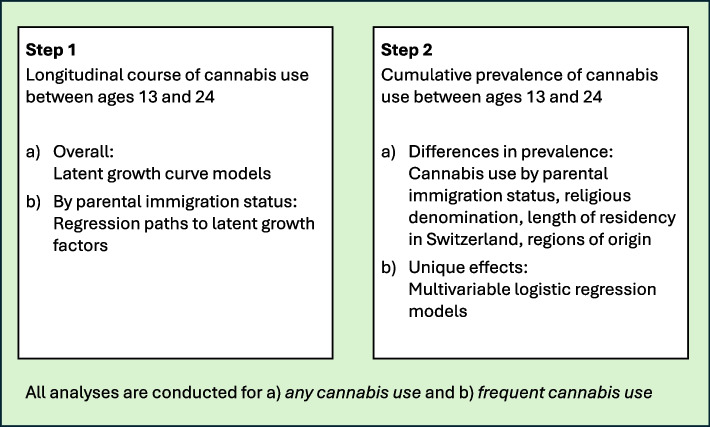
Steps of analyses

Figure 1 provides a summary of the steps of our analyses. First, to examine whether the longitudinal trajectory of cannabis use between the ages of 13 and 24 years differed between the groups with one, two, or no immigrant parents, we specified latent growth curve models. We specified the age-13-assessment as the intercept and tested the linear and quadratic latent slopes to identify the average shape of the cannabis use trajectory in our sample. We subsequently tested for associations between the immigration status of the participants’ parents and the latent growth factors. Second, we examined group differences in the cumulative prevalence of cannabis use from ages 13 to 24. We also tested the potential mechanisms underlying these differences by specifying multivariable regression models that included the immigration status, and, simultaneously, religious denomination, length of residency in Switzerland (categorical measure with a reference category: nonimmigrant primary caregiver), and specific regions of origin as predictors (more details on model specifications are provided in the “Results” section).

In all analyses, we specified models for two outcomes: (a) any cannabis use, and (b) frequent cannabis use. All models were specified in MPlus V8 (Muthén & Muthén, 2017) and adjusted for participant sex assigned at birth, the primary caregiver’s age, and the household’s socio-economic status. For the descriptive statistics, we performed group comparisons based on the observed data; in the multivariable models, we used multiple imputation to address missing data. The latter is a gold standard in longitudinal research and minimizes potential bias due to selective attrition mechanisms (Enders, 2013).

The z-proso study reached its highest participant rates when the adolescents were 15 years old (Eisner et al., 2018). Therefore, we used the age-15 assessment to examine whether migration background was associated with attrition in early adulthood (age 24). Of those who participated at age 15 (*n* = 1446), 79% also participated at age 24 (*n* = 1144). Attrition was higher (*p* < 0.001) in the group with two immigrant parents (26%) than in the groups with two nonimmigrant parents (15%) and one immigrant parent (16%). The multiple imputation models included the same variables as the analytic models. For cumulative cannabis use, we imputed the five cannabis variables assessed from ages 13 to 24 and then created the cumulative score based on the complete data. We imputed 10 datasets and pooled the results (Rubin, 1987).

## Results

Table 1 shows descriptive statistics for the three groups defined by parental immigration status, including the socio-demographic and immigration-related variables. Families with two nonimmigrant parents had the highest socio-economic status, whereas those with two immigrant parents had the lowest status. The groups with one and two immigrant parents differed regarding the parents’ regions of origin. In the group with two immigrant parents, the latter were more often born in former Yugoslavia and Asia than those in the group with one immigrant parent, whereas the proportion of parents born in Africa or Latin America was higher in the one immigrant parent group.

**Table 1:** Socio-demographic and immigration-related characteristics of the three analytic groups

	1. Both parents born in Switzerland	2. One parent born abroad	3. Two parents born abroad	Significant group differences (*p* < 0.05)
*n*	341	388	716	
Age: M (SD)				
- First assessment	13.65 (0.36)	13.64 (0.37)	13.69 (0.37)	--
- Second assessment	15.43 (0.36)	15.42 (0.37)	15.45 (0.36)	--
- Third assessment	17.43 (0.35)	17.41 (0.38)	17.47 (0.38)	2/3
- Fourth assessment	20.54 (0.36)	20.55 (0.40)	20.61 (0.39)	1/3, 2/3
- Fifth assessment	23.95 (0.41)	23.95 (0.39)	23.98 (0.38)	--
Sex (as recorded at birth): % (n/n)				
- Male	56.6 (193/341)	49.7 (193/388)	50.3 (360/716)	--
- Female	43.4 (148/341)	50.3 (195/388)	49.7 (356/716)	--
Socio-economic status (ISEI): M (SD)	55.72 (15.88)	52.92 (17.36)	36.83 (17.78)	1/2, 1/3, 2/3
Region of origin (parent pairs): % (n/n)				
- Switzerland	100 (341/341)	--	--	n/a
- Former Yugoslavia	--	--	33.0 (234/710)	n/a
- Europe	--	--	24.4 (173/710)	n/a
- Asia	--	--	25.6 (182/710)	n/a
- Africa	--	--	5.1 (36/710)	n/a
- Latin America	--	--	3.2 (23/710)	n/a
- Other and mixed, two immigrant parents	*--*	--	8.7 (62/710)	n/a
- Mixed, one nonimmigrant parent	--	100 (383/383)	--	n/a
Region of origin (any parent): % (n/n)				
- Switzerland	100 (341/341)	100 (383/383)	0 (0/710)	2/3^a^
- Former Yugoslavia	--	7.6 (29/383)	35.2 (250/710)	2/3^a^
- Europe (EU and other)	--	46.0 (176/383)	31.4 (223/710)	2/3^a^
- Asia	--	17.5 (67/383)	29.3 (208/710)	2/3^a^
- Africa	--	10.2 (39/383)	6.8 (48/710)	--^a^
- Latin America	--	13.8 (53/383)	5.1 (36/710)	2/3^a^
- Other	--	5.0 (19/383)	1.0 (7/710)	2/3^a^
Religious denomination: % (n/n)				
- None	28.5 (97/340)	26.4 (102/386)	8.8 (62/707)	1/3, 2/3
- Christian	67.9 (231/340)	60.6 (234/386)	44.3 (313/707)	1/2, 1/3, 2/3
- Muslim	0.9 (3/340)	10.9 (42/386)	35.8 (253/707)	1/2, 1/3, 2/3
- Hindu	--	0.3 (1/386)	9.8 (69/707)	1/3, 2/3
- Other	2.4 (8/340)	1.8 (7/386)	1.3 (9/707)	--
Length of residency in Switzerland at study onset: % (n/n)				
- 0–5 years	--	7.5 (19/252)	9.1 (43/473)	--
- 6–10 years	--	32.1 (81/252)	35.1 (166/473)	--
- 11–15 years	--	19.4 (49/252)	33.6 (159/473)	2/3
- 16–20 years	--	17.1 (43/252)	15.2 (72/473)	--
- 21 years and more	--	23.8 (60/252)	7.0 (33/473)	2/3

*ISEI* International Socio-Economic Index; *n/a* not applicable

^a^ Significance test was conducted only for the comparison of groups 2 vs. 3

Those with one or two nonimmigrant parents were mostly affiliated with the Christian religion or had no religious affiliation, whereas those with two immigrant parents were mostly affiliated with the Christian or Muslim religions. The group with two immigrant parents was the only one with a substantial proportion of participants being affiliated with the Hindu religion. Finally, a medium parental length of residency in Switzerland (11–15 years) was more common in the group with two immigrant parents than in the group with one immigrant parent, whereas in the latter, the longest duration of residency (21 years and more) was more common.

### Longitudinal Course of Cannabis Use by Migration Status and Sex

**Fig. 2:**
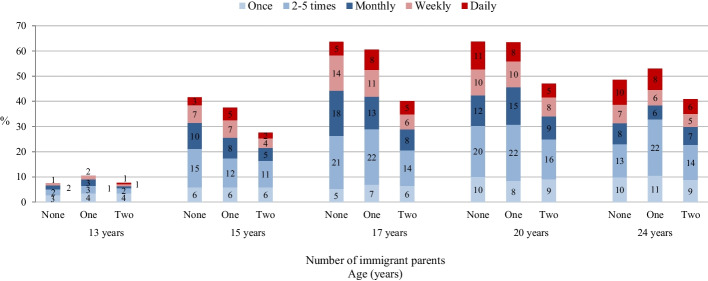
Past-year prevalence and frequency of cannabis use by parental immigration status. *Note*. Frequent cannabis use categories in red (dark and light)

Figure 2 shows that, across all groups, the prevalence and frequency of cannabis use increased from the early teens to age 20 and then decreased slightly until age 24. A latent growth curve model excluding predictor variables revealed an overall curvilinear increase in any cannabis use (Table 2, Model A). A model including predictors showed that males generally reported higher initial rates and flatter subsequent increases in cannabis use. In addition, there were immigration effects: At age 13, the odds of any cannabis use were similarly low across all immigration groups; but cannabis use subsequently increased more rapidly among those with two nonimmigrant parents than among those with two immigrant parents, as indicated by a negative association between having two immigrant parents and the linear growth factor. No group difference emerged between those with two nonimmigrant parents and those with one immigrant parent. Finally, we found a positive association between having two immigrant parents and the quadratic growth factor, which indicates that, among those with two nonimmigrant parents (i.e., the reference group), the rapid initial increase in cannabis use from early adolescence to age 20 was followed by a particularly steep decline to age 24.

**Table 2:** Results from latent growth curve models, outcomes: growth factors of cannabis use between ages 13 and 24

Growth factors/Predictors	Estimate	*p*
**Model A: **Any cannabis use		
Means of growth factors^a^		
- Linear growth factor	**1.26**	< 0.001
- Quadratic growth factor	**−0.09**	< 0.001
Predictors of growth factors: unstandardized regression coefficients		
Intercept (Age 13)		
- Sex: male (ref.: female)	**1.84**	< 0.001
- Socio-economic status (ISEI)	0.01	0.067
- One parent born abroad (ref.: two nonimmigrant parents)	0.39	0.213
- Two parents born abroad (ref.: two nonimmigrant parents)	0.12	0.689
Linear growth factor		
- Sex: male (ref.: female)	**−0.39**	0.001
- Socio-economic status (ISEI)	0.01	0.084
- One parent born abroad (ref.: two nonimmigrant parents)	−0.24	0.101
- Two parents born abroad (ref.: two nonimmigrant parents)	**−0.48**	< 0.001
Quadratic growth factor		
- Sex: male (ref.: female)	**0.04**	< 0.001
- Socio-economic status (ISEI)	0.00	0.372
- One parent born abroad (ref.: two nonimmigrant parents)	0.03	0.055
- Two parents born abroad (ref.: two nonimmigrant parents)	**0.04**	0.004
**Model B:** Frequent cannabis use		
Means of growth factors^a^		
- Linear growth factor	**1.24**	0.003
- Quadratic growth factor	**−0.16**	0.042
Predictors of growth factors: unstandardized regression coefficients		
Intercept (Age 13)		
- Sex: male (ref.: female)	**2.32**	< 0.001
- Socio-economic status (ISEI)	0.01	0.416
- One parent born abroad (ref.: two nonimmigrant parents)	0.51	0.326
- Two parents born abroad (ref.: two nonimmigrant parents)	0.01	0.977
Linear growth factor		
- Sex: male (ref.: female)	−0.38	0.095
- Socio-economic status (ISEI)	0.00	0.813
- One parent born abroad (ref.: two nonimmigrant parents)	−0.07	0.762
- Two parents born abroad (ref.: two nonimmigrant parents)	−0.28	0.184
Quadratic growth factor		
- Sex: male (ref.: female)	**0.07**	0.003
- Socio-economic status (ISEI)	0.00	0.909
- One parent born abroad (ref.: two nonimmigrant parents)	−0.00	0.846
- Two parents born abroad (ref.: two nonimmigrant parents)	0.01	0.549

*ISEI* International Socio-Economic Index

^a^ The means of the growth factors were estimated in a model excluding predictor variables

*n* = 1445, 10 imputed data sets, results pooled. Bold print indicates estimates with *p* < 0.05

**Fig. 3:**
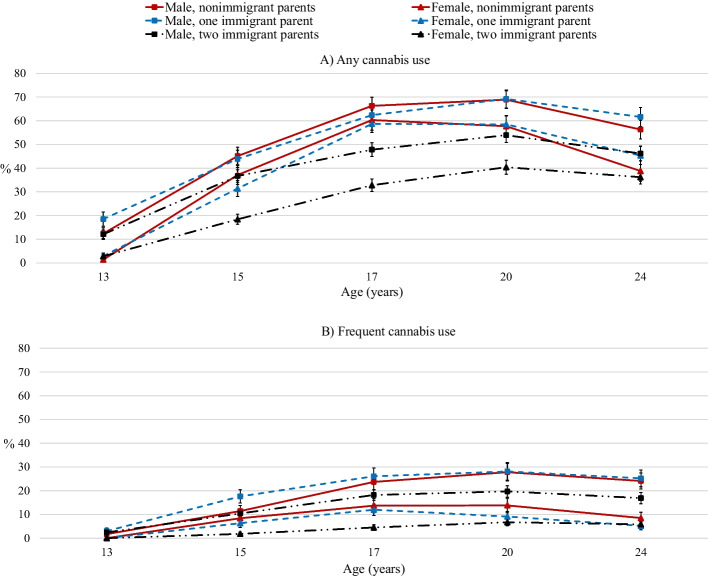
Past-year prevalence of any cannabis use and frequent cannabis use among male and female offspring by parental immigration status

Group-differences in cannabis use were especially large during the participants’ late teens and early twenties and smaller in their mid-twenties (see Fig. 3 for an illustration by group and sex). Notably, during the late teens and early twenties, the immigrant effect was overriding the sex effect (i.e., non-immigrant females’ cannabis use prevalence exceeded the prevalence of cannabis use in immigrant males, see Fig. 3, Panel A). Regarding the longitudinal trajectory of *frequent* cannabis use, no differences by immigration groups emerged (Table 2, Model B), and male adolescents generally had higher rates of frequent cannabis use than females (Fig. 3, Panel B).

### Cumulative Prevalence of Cannabis Use by Migration Status and Sex

**Fig. 4:**
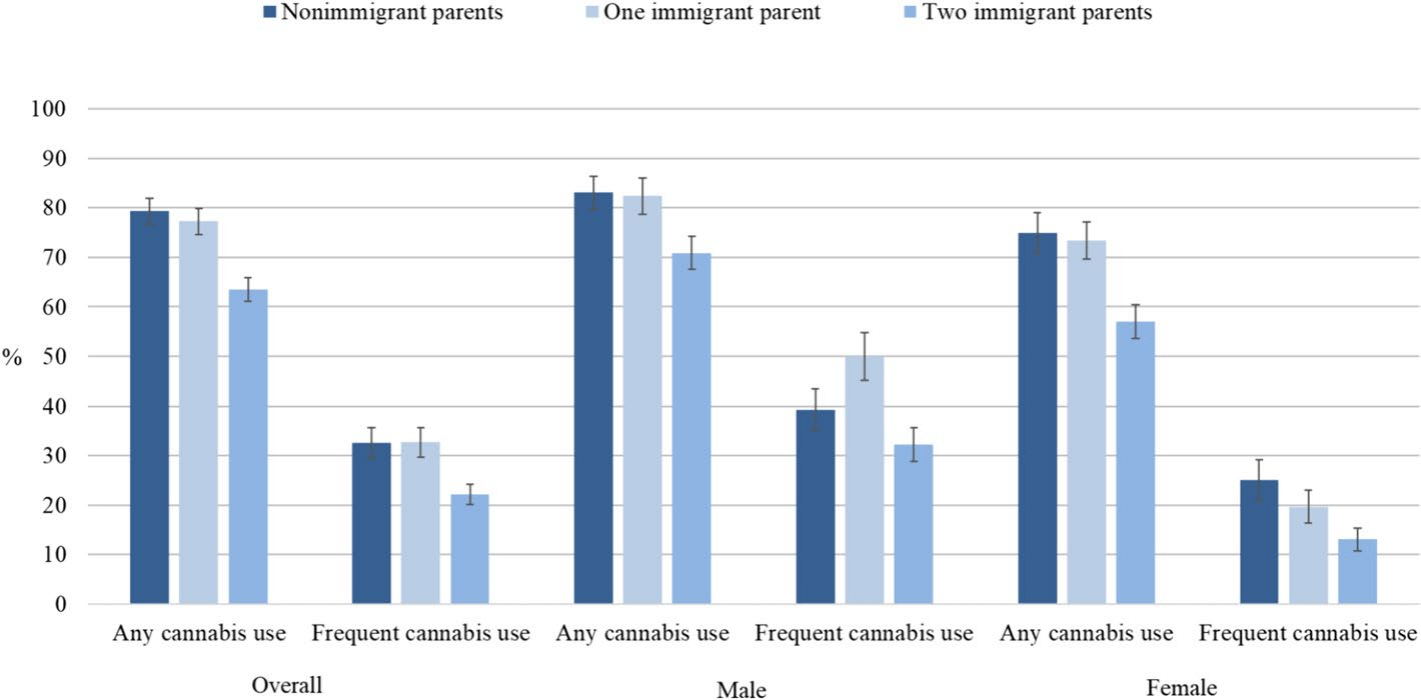
Cumulative past-year prevalence of any cannabis use and frequent cannabis use between the ages of 13 and 24 years by parental immigration status. *Note.* The following group differences were significant (*p* < 0.05): 1. Overall, any cannabis use: nonimmigrant parents vs. two immigrant parents (*p* < 0.001), one immigrant parent vs. two immigrant parents (*p* < 0.001). 2. Overall, frequent cannabis use: nonimmigrant parents vs. two immigrant parents (*p* = 0.003), one immigrant parent vs. two immigrant parents (*p* = 0.003). 3. Male, any cannabis use: nonimmigrant parents vs. two immigrant parents (*p* = 0.013), one immigrant parent vs. two immigrant parents (*p* = 0.027). 4. Male, frequent cannabis use: one immigrant parent vs. two immigrant parents (*p* = 0.003). 5. Female, any cannabis use: nonimmigrant parents vs. two immigrant parents (p = 0.001), one immigrant parent vs. two immigrant parents (p = 0.003). 6. Female, frequent cannabis use: nonimmigrant parents vs. two immigrant parents (p = 0.007)

Adolescents with two nonimmigrant parents and those with one immigrant parent had a higher cumulative prevalence of *any cannabis use* between ages 13 and 24 than those with two immigrant parents (Fig. 4). A similar pattern emerged for *frequent cannabis use*. Both male and female adolescents with two nonimmigrant parents and those with one immigrant parent reported more cannabis use than those with two immigrant parents. Regarding frequent cannabis use, sex-specific differences were less consistent.

### Cumulative Prevalence and Mechanisms: Bivariate Associations

**Fig. 5:**
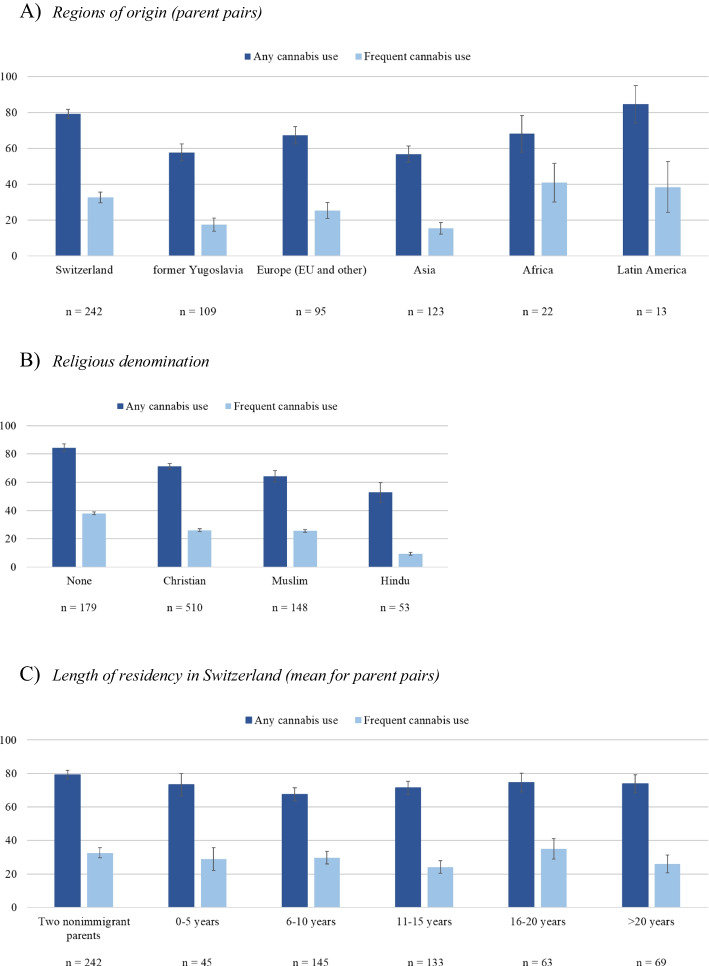
Cumulative prevalence of cannabis use (13–24 years) by parents’ regions of origin (illustration limited to families where both parents were born in the same region), religious denomination, and length of residency

Youth with two nonimmigrant parents reported any cannabis use more often than those with parent pairs from any other regions of origin, except Latin America (Fig. 5a). Those without a religious denomination had a higher cumulative prevalence of any cannabis use than those with Christian, Muslim, or Hindu denominations (Fig. 5b). For length of residency, the cumulative prevalence of any cannabis use was especially low among those whose parents had migrated to Switzerland about six to 10 years before the participants’ school enrollment (Fig. 5c). For *frequent cannabis use*, the patterns looked partly similar (e.g., any religious denomination was associated with lower rates of frequent cannabis use than no religious denomination) and partly a bit less systematic (e.g., variations by length of residency seemed to be more random; see Fig. 5a–c).

### Cumulative Prevalence and Mechanisms: Unique Effects

In a first multivariable regression model (model A), we tested whether differences by immigration group remained significant when including religious denominations and length of residency as predictors in the same model (regions of origin were not included because, when being included in the same model as the dummy variable indicating two immigrant parents, the region of origin dummy variables provide redundant information). The results revealed that all three major religious denominations were uniquely associated with a lower likelihood of *any cannabis use* between the ages of 13 and 24 years compared to no religious denomination (Table 3). There were no additional effects of immigration group, indicating that the immigration effect could be fully explained by group differences in religious denomination.

**Table 3:** Multivariable regression model, outcome: any cannabis use between the ages of 13 and 24 years

Predictors	Odds ratios	95% confidence intervals	
		Lower bound	Upper bound
**Model A: **Comparison of no, one, and two immigrant parents			
Adolescent sex: male (ref.: female)	**1.82**	1.41	2.36
Primary caregiver’s age	**1.03**	1.00	1.06
Household socio-economic status (ISEI)	**1.01**	1.00	1.02
One immigrant parent (ref.: two nonimmigrant parents)	1.21	0.78	1.89
Two immigrant parents (ref. : two nonimmigrant parents)	0.96	0.63	1.47
Religious denomination (ref.: none)			
- Christian	**0.65**	0.45	0.94
- Muslim	**0.51**	0.32	0.80
- Hindu	**0.39**	0.21	0.73
- Other	1.28	0.35	4.71
Length of residency in Switzerland (ref.: nonimmigrant primary caregiver)			
- 0-5 years	0.97	0.68	1.39
- 6-10 years	0.80	0.58	1.12
- 10-15 years	1.13	0.80	1.59
- 16-20 years	1.00	0.67	1.49
- 21 years and more	0.95	0.56	1.61
**Model B:** Specific regions of origin (parent pairs)			
Adolescent sex: male (ref.: female)	**1.72**	1.33	2.23
Primary caregiver’s age	**1.03**	1.00	1.06
Household socio-economic status (ISEI)	**1.01**	1.00	1.02
One immigrant parent (ref.: two nonimmigrant parents)	0.99	0.56	1.75
Two immigrant parents: region of origin (ref.: two nonimmigrant parents)			
- Former Yugoslavia	**0.52**	0.29	0.94
- Europe	0.83	0.46	1.49
- Asia	**0.54**	0.30	0.99
- Africa	0.55	0.23	1.29
- Latin America	2.18	0.58	8.20
- Mixed	1.61	0.66	3.96
Religious denomination (ref.: none)			
- Christian	**0.68**	0.47	1.00
- Muslim	0.66	0.40	1.10
- Hindu	**0.47**	0.22	1.00
- Other	1.41	0.40	4.94
Length of residency in Switzerland (ref.: primary caregiver nonimmigrant)			
- 0-5 years	1.04	0.55	1.97
- 6-10 years	1.11	0.71	1.73
- 10-15 years	1.40	0.87	2.26
- 16-20 years	1.17	0.78	1.73
- 21 years and more	0.91	0.49	1.69

*n* = 1445, 10 imputed data sets, results pooled. Bold print indicates significant associations. Note that the values are rounded, which is why in some places bold print of odds ratios indicates significance although the – rounded - confidence interval includes the value 1

In a second model (model B), we replaced the “two immigrant parents” dummy with the variables representing specific regional origins of these parent pairs. Having two parents from former Yugoslavia or Asia was associated with lower odds of any cannabis use than having two nonimmigrant parents. Christian and Hindu affiliations were still uniquely associated with lower odds of cannabis use than having no religious affiliation, whereas the effect of a Muslim affiliation was nonsignificant.

The multivariable models also revealed that youth with a Hindu affiliation had lower odds of *frequent cannabis use* than those without any religious denomination (Table 4, model A). However, the effect was nonsignificant in model B (i.e., when including specific regions of origin). The group with two immigrant parents from different regions (i.e., mixed) reported a relatively high prevalence of frequent cannabis use, but due to small group sizes we were not able to explore this further. The effects of other predictors on frequent cannabis use were nonsignificant.

**Table 4:** Multivariable regression model, outcome: any frequent cannabis use between the ages of 13 and 24 years

Predictors	Odds ratios	95% confidence intervals	
		Lower bound	Upper bound
**Model A: **Comparison of no, one, and two immigrant parents			
Adolescent sex: male (ref.: female)	**3.90**	2.97	5.13
Primary caregiver’s age	1.00	0.97	1.03
Household socio-economic status (ISEI)	1.00	0.99	1.01
One immigrant parent (ref.: two nonimmigrant parents)	1.38	0.89	2.15
Two immigrant parents (ref.: two nonimmigrant parents)	1.04	0.66	1.65
Religious denomination (ref.: none)			
- Christian	0.77	0.56	1.05
- Muslim	0.70	0.45	1.08
- Hindu	**0.36**	0.18	0.74
- Other	2.04	0.83	4.97
Length of residency in Switzerland (ref.: nonimmigrant primary caregiver)			
- 0–5 years	0.84	0.59	1.20
- 6–10 years	1.02	0.72	1.46
- 10–15 years	0.91	0.64	1.30
- 16–20 years	1.17	0.81	1.69
- 21 years and more	0.80	0.48	1.33
**Model B: **Specific regions of origin (parent pairs)			
Adolescent sex: male (ref.: female)	**3.80**	2.91	4.95
Primary caregiver’s age	1.00	0.98	1.03
Household socio-economic status (ISEI)	1.00	0.99	1.01
One immigrant parent (ref.: two nonimmigrant parents)	1.28	0.74	2.20
Two immigrant parents: region of origin (ref.: two nonimmigrant parents)			
- Former Yugoslavia	0.69	0.38	1.26
- Europe	1.03	0.59	1.82
- Asia	0.75	0.38	1.50
- Africa	1.35	0.53	3.42
- Latin America	2.55	0.99	6.57
- Mixed	**2.28**	1.13	4.61
Religious denomination (ref.: none)			
- Christian	0.84	0.60	1.16
- Muslim	0.85	0.53	1.39
- Hindu	0.49	0.21	1.16
- Other	2.05	0.82	5.12
Length of residency in Switzerland (ref.: primary caregiver nonimmigrant)			
- 0–5 years	0.96	0.50	1.85
- 6–10 years	1.06	0.68	1.63
- 10–15 years	0.87	0.51	1.49
- 16–20 years	1.00	0.69	1.46
- 21 years and more	0.74	0.40	1.38

*n* = 1445, 10 imputed data sets, results pooled. Bold print indicates significant associations

## Discussion

Our study is among the first to explore the differences between immigrant and nonimmigrant youth’s cannabis use from a developmental perspective. Consistent with findings from the USA and other Western European countries (Amundsen et al., 2005; Gfroerer & Tan, 2003), immigrant youth in our Swiss study reported lower rates of cannabis use compared to their peers with at least one nonimmigrant parent. Going beyond the mostly cross-sectional prior evidence (Campisi et al., 2017; Hüsler & Werlen, 2010; Lukash & Killias, 2018), our study revealed that differences in any cannabis use between immigrant and nonimmigrant youth emerge after early adolescence (i.e., after age 13) and are especially large between mid-adolescence and the early twenties. Specific cultural and religious backgrounds seem to account for the especially low rates of cannabis use in youth with two immigrant parents.

The curvilinear increase in any and frequent cannabis use from the early teens to the mid-twenties in our sample is consistent with evidence from the USA (Copeland et al., 2017), which did not distinguish groups with immigrant and nonimmigrant parents. Our finding of an especially steep increase in cannabis use among adolescents with two nonimmigrant parents suggests that prevention mechanisms need to begin in early adolescence, particularly for males. For youth with two immigrant parents, prevention programs could still be effective during mid- and late adolescence, given the slower initial increase in cannabis use during adolescence in this group. The larger gap in the prevalence of any cannabis use between nonimmigrant and immigrant youth in adolescence, compared to early adulthood, likely indicates that many nonimmigrant adolescents experiment only temporarily with using cannabis. However, an early onset of substance use increases the risk of severe mental and physical health impairments, including damage to the developing adolescent brain and, for example, substance use disorder in adulthood (Hall et al., 2016; Jordan & Andersen, 2017; Shanahan et al., 2021; Volkow et al., 2014; Vonmoos et al., 2013).

Considering recent demographic trends and established ages of onset, a recent simulation study predicted an increase in the lifetime prevalence of cannabis use in the Swiss population from one-third in 2015 to one-half in 2045 (Vogel et al., 2019). The high rates of cannabis use in all groups considered in our study suggest that in the city of Zurich, the prevalence of cannabis use among 24-year-olds already far exceeds the projected rates from that previous study in both nonimmigrant and immigrant subpopulations. Although we found that prevalence was highest among nonimmigrant males, and lowest among immigrant females, more than one in two immigrant females reported cannabis use at least once between the ages of 13 and 24.

Previous research has not explicitly investigated differences in *frequent* cannabis use by immigration status (Campisi et al., 2017; Hüsler & Werlen, 2010). In our study, the immigrant effect was less consistent for frequent use than for any use. This is in line with previous evidence on alcohol use (Amundsen et al., 2005) and suggests that immigrant youth may have a higher threshold for initiating cannabis use but not for progressing to more regular use. Frequent use, which might occur to cope with stress and signal an adolescent’s increased risk of cannabis use disorder (Bottorff et al., 2009; Copeland et al., 2017), may have more pronounced biological than cultural underpinnings.

What factors could explain the observed group differences in cannabis use prevalence? In many of the regions where our participants’ parents came from, cannabis use was likely viewed as more harmful and less normative than in Switzerland (Andersson et al., 2009; Degenhardt et al., 2008; Piontek et al., 2013). Immigrant parents may transmit such views and values to their children. Second, immigrant parents may perceive their family’s legal status in the new country as fragile. Accordingly, they may enforce stricter rules about substance use, and allow their adolescents fewer liberties compared to the many liberties that Swiss adolescents typically have.

Our findings were consistent with some previous research in that Christian and Muslim youth were less likely to consume cannabis than youth without a religious affiliation (Abebe et al., 2015; Amundsen et al., 2005). Our study had a large enough Hindu subsample to reveal that this group, which had previously often been combined with other religions into one group, had an especially low risk of cannabis use. Notably, immigrant youth were more likely than nonimmigrant youth to have a religious affiliation, and Muslim or Hindu affiliations specifically; and, indeed, religion accounted for some of the differences in cannabis use between immigrant and nonimmigrant youth in our sample. Importantly, religious affiliations are often correlated with specific regional origins. For example, in our study, a Muslim denomination was mostly associated with having at least one immigrant parent born in former Yugoslavia (> 50%) or Asia (> 30%), and a Hindu denomination was exclusively associated with parental birth in Asia. Given that some religious affiliations were not associated with a decreased likelihood of cannabis use once that specific regions of parental origin were included in the same model, future research should aim to further disentangle the effects of religious versus regional backgrounds on young people’s cannabis use.

The low odds of cannabis use among children whose parents were born in former Yugoslavia or Asia mirror findings from international comparisons of adolescent cannabis use. For example, based on self-reports collected in 2002, a study found that adolescents living in former Yugoslavia and those in Switzerland represented the two extremes of cannabis use prevalence: it was very low in former Yugoslavia and very high in Switzerland (Ter Bogt et al., 2006). In that study, mechanisms underlying regional differences in cannabis use rates included a country’s wealth, perceived availability of cannabis, and a specific “drug culture” (i.e., being surrounded by a considerable community of young people using cannabis). Our study shows that, even after immigrating to a wealthy country with high overall cannabis use rates and high actual availability of cannabis, parents who have originally been socialized in a country with low cannabis use rates were likely to raise their children in ways that these children abstained from cannabis more often than their non-immigrant peers.

Lack of evidence of pure acculturation effects (as indicated by length of residency in Switzerland), which have been documented by others (Amundsen et al., 2005; Lukash & Killias, 2018), may be due to rather small group sizes and potential overlap of the length of residency and specific waves of immigration due to war, economic or environmental crises, and persecution of religious minorities in particular regions at certain times. It is also noteworthy that longer residencies in Switzerland were more prevalent among families with one immigrant parent than among those with two immigrant parents. There may have been some overlap (e.g., in terms of acculturation) between long residencies in Switzerland and pairing up with a nonimmigrant person in explaining the indifference in cannabis use rates between youth with one and two nonimmigrant parents.

### Limitations and Suggestions for Future Research

Limitations of our study include, first, that the findings may not generalize to other regions with different shares of immigrant and nonimmigrant youth and where cannabis may not be as easily accessible as in Switzerland and, specifically, Zurich. Second, self-reports of cannabis use could be attenuated due to social desirability or recall bias; however, in a previous investigation based on the z-proso sample and including hair toxicology analyses, no association between parental immigration status and the risk of underreporting substance use emerged (Steinhoff et al., 2023). Third, we focused on social-structural factors related to immigration and their association with cannabis use. Further research is needed to compare the impact of these factors to that of psychological factors, other health related factors, and social experiences (e.g., parenting), which have previously been associated with substance use, including in z-proso (Rodríguez-Ruiz, Zych, Ribeaud, et al., 2023; Shanahan et al., 2021; Steinhoff et al., 2022). Indeed, there could be a selection effect of immigration. For example, it is possible that in at least a segment of immigrants, it is individuals with better health who migrate to Switzerland (Markides & Rote, 2015).

It is important to note that our sample included mostly second-generation immigrant adolescents, and a relatively small proportion of youth who had, themselves, experienced immigration. Previous research revealed significant differences in substance use by generational status and interactions between generational status and other socio-demographic variables as well as country of origin (Cook et al., 2021). These differences need to be explored further regarding the longitudinal course of adolescent substance use in future research. Generally, within-group differences in the risk of cannabis and other substance use among both immigrant and nonimmigrant youth should be further explored in future research to develop well-tailored prevention programs.

Due to otherwise small group sizes, we combined several countries to represent certain regions or continents and future research should aim to provide a more nuanced perspective on cultural backgrounds. Furthermore, while our focus was on the role of religious denominations in adolescence in predicting cannabis use, future research may consider that religious denominations could change over time and investigate further how such changes are related to substance use. Finally, future research is needed to explore the intersection of migration backgrounds, sex/gender, and specific cultural/socio-economic contexts further, since the higher prevalence of cannabis use in male adolescents documented here contrasts with some recent evidence from other countries (e.g., USA or Spain), where the prevalence of adolescent substance use did not differ between sexes or was higher among females (Harlow et al., 2024; Rodríguez-Ruiz, Zych, Llorent, et al., 2023). The impact of gender (roles) should also be explored further with regard to families with one immigrant parent; for example, the impact of fathers versus mothers with an immigration background on adolescent cannabis use may differ.

## Conclusions and Implications

Our longitudinal cohort study documents that differences in cannabis use between immigrant and nonimmigrant youth change across the adolescent period. The especially large group differences between mid-adolescence and the early twenties may indicate different motivations and legitimizations for cannabis use. The latter need to be examined in future research to possibly be considered in the development of prevention and intervention programs. Such programs may be especially promising in early to mid-adolescence and should consider the role of cultural and religious backgrounds of young people. Despite the differences between immigrant and nonimmigrant youth, however, prevention and intervention programs generally need to target all youth, given the high prevalence of any and frequent cannabis use in all groups.

## Data Availability

The data used in this study are available from the corresponding author upon request.
